# Leukostasis Presenting as a STEMI: A Case Report

**DOI:** 10.51894/001c.166036

**Published:** 2026-08-01

**Authors:** Hope Welter, Parker Lehmann, Casey Reulbach, Christopher Szyarto

**Affiliations:** 1 A.T. Still University

## 131

### Introduction

Leukostasis is a hematologic emergency as a result of hyperleukocytosis, a condition most commonly associated with hematologic malignancies such as acute myeloid leukemia (AML). The typical presentation includes respiratory distress and/or neurologic dysfunction.

### Case presentation

The case discussed demonstrates a rare presentation of leukostasis as chest pain with ST-elevation myocardial infarction (STEMI) on electrocardiogram in a patient with a history of cardiovascular disease. The patient underwent cardiac catheterization which was unremarkable for clot burden. Further workup showed hyperleukocytosis, with resultant bone marrow biopsy making the diagnosis of AML. The patient experienced symptom resolution after leukapheresis. They received inpatient induction therapy for their newly diagnosed AML with subsequent ongoing outpatient management.

### Discussion

Early recognition of this atypical presentation is crucial to reduce or prevent irreversible organ damage by initiating cytoreductive therapy without delay.

### Conclusion

In patients presenting with STEMI, leukostasis should be considered in the differential diagnoses, even in those with cardiac risk factors.

